# Effective selection of informative SNPs and classification on the HapMap genotype data

**DOI:** 10.1186/1471-2105-8-484

**Published:** 2007-12-20

**Authors:** Nina Zhou, Lipo Wang

**Affiliations:** 1 Electrical and Electronic Engineering, Nanyang Technology University, Block S1, 50 Nanyang Avenue, 639798 Singapore

## Abstract

**Background:**

Since the single nucleotide polymorphisms (SNPs) are genetic variations which determine the difference between any two unrelated individuals, the SNPs can be used to identify the correct source population of an individual. For efficient population identification with the HapMap genotype data, as few informative SNPs as possible are required from the original 4 million SNPs. Recently, Park *et al.* (2006) adopted the nearest shrunken centroid method to classify the three populations, i.e., Utah residents with ancestry from Northern and Western Europe (CEU), Yoruba in Ibadan, Nigeria in West Africa (YRI), and Han Chinese in Beijing together with Japanese in Tokyo (CHB+JPT), from which 100,736 SNPs were obtained and the top 82 SNPs could completely classify the three populations.

**Results:**

In this paper, we propose to first rank each feature (SNP) using a ranking measure, i.e., a modified t-test or F-statistics. Then from the ranking list, we form different feature subsets by sequentially choosing different numbers of features (e.g., 1, 2, 3, ..., 100.) with top ranking values, train and test them by a classifier, e.g., the support vector machine (SVM), thereby finding one subset which has the highest classification accuracy. Compared to the classification method of Park *et al.*, we obtain a better result, i.e., good classification of the 3 populations using on average 64 SNPs.

**Conclusion:**

Experimental results show that the both of the modified t-test and F-statistics method are very effective in ranking SNPs about their classification capabilities. Combined with the SVM classifier, a desirable feature subset (with the minimum size and most informativeness) can be quickly found in the greedy manner after ranking all SNPs. Our method is able to identify a very small number of important SNPs that can determine the populations of individuals.

## Background

When any one single nucleotide of *A*, *T*, *C *and *G *in the genome sequence is replace by one of any other 3 nucleotide, e.g., from AAATCCGG to AAATTCGG, we call this single base variation (C ⇒ T) as a single nucleotide polymorphism (SNP). It has the following three characteristics [1]: 1) very common in the human genome (a SNP occurs every 100 to 300 bases along the 3-billion-base human genome); 2)among the SNPs, two of every three SNPs are the variations from cytosine (C) to thymine (T); 3) very stable from generation to generation. Due to these characteristics, much research on SNPs has been developed, such as using SNPs to study the association of sequence variation [2-5] and to do population classification [6,7].

In association studies [2-5], informative SNPs were usually selected based on certain correlation measures and therefore could represent other SNPs in the close proximity. For example, Bafna et al. [2] and Halldrsson et al. [3] proposed to select a subset of tag SNPs with the minimum size and highest informativeness value calculated from a self-defined informativeness measure, which evaluates how well a single SNP or a set of SNPs predict another single SNP or another set of SNPs within the neighborhoods. Eran et al. [4] proposed to select the informative SNPs with the maximum prediction accuracy, which is obtained from a prediction accuracy measure evaluating how well the value of an SNP is predicted by the values of only two closest tag SNPs. Phuong et al. [5] proposed the method of selecting informative SNPs by removing redundant features. Redundancy was measured by feature similarity between two features, i.e., the linkage disequilibrium (LD) measure *γ*^2 ^[5].

In population studies, the selection of informative SNPs should be based on their population classification capability. Related research, such as selecting genetic markers with highest informativeness for inference of individual ancestry [8], selecting informative marker panels for population assignment [6] and detecting ethnically variant SNPs [7], has already been explored. Rosenberg et al. [8] proposed to use the informativeness for assignment (*I*_*n*_) to measure the ability of each genetic loci or marker (feature) to infer individuals' ancestry, which was proved to be similar to the F-statistics measure [8]. In [6], Rosenberg et al. proposed the univariate, greedy, and maximum algorithms to select marker panels. The three algorithms were realized through a given performance function, e.g., the optimal rate of correct assignment (ORCA) [8], which measures the probability of correctly assigning an individual to the population from which the genotype of the individual has originated with the greatest possibility. The application of the algorithms on eight species was effective. Very recently, Park et al. developed a systematic approach based on nearest shrunken centroid (NSCM) method [9] to identify ethnically variant SNPs. According to [9], they calculated a shrunken value for each SNP of each class, and compared each SNP's shrunken value for different classes to determine the SNP's classification capability. The less the difference among the SNP's shrunken values for different classes, the less important the SNP for classifying the three different ethnic groups (classes) [10], i.e., CEU, YRI and JPT+CHB. 100,736 SNPs were obtained and the top 82 SNPs were able to completely classify the three populations.

In this paper, we propose to firstly rank SNPs according to a feature importance ranking measure, i.e., a modified t-test or F-statistics, where the higher the ranking value, the stronger the corresponding classification power. Then, from the ranking list, we sequentially choose different numbers of top ranked SNPs, e.g., 1, 2, 3, ..., 20 and so on, test them through a classifier, e.g., the support vector machine (SVM) [11,12] and determine the SNP subset which has the highes classification accuracy. This process is repeated 30 times. Finally, we locate those important SNPs who always have top ranking values according to SNP subsets obtained from 30 simulations.

## Results and discussion

**Figure 1:**
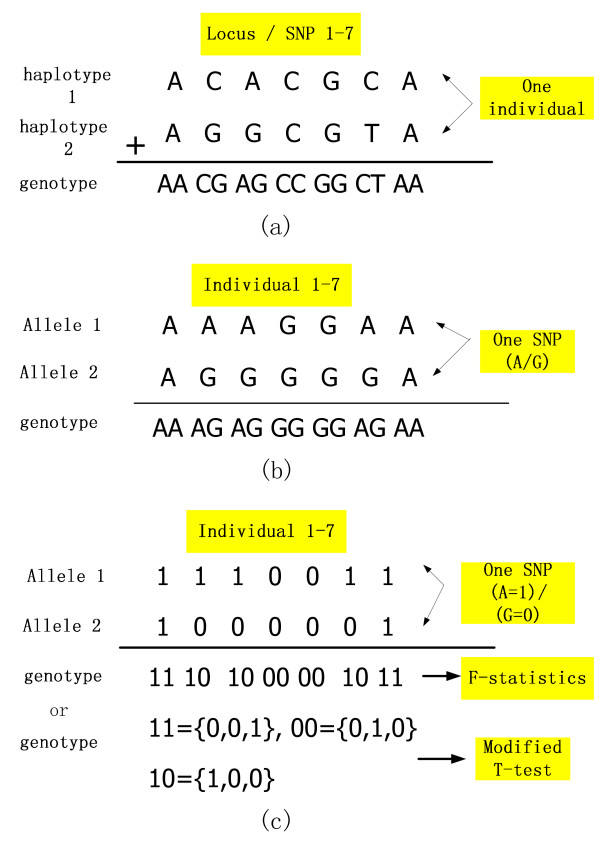
**Example of haplotypes and genotypes**. (a). The haplotype and genotype formats of one individual; (b). Different nominal values (genotype format) of one SNP for different individuals; (c). Numerical values of one SNP for different individuals in (b), in which the first transformation is for the F-Statistics algorithm, the second transformation in vector format is for the modified t-test algorithm.

The international HapMap Project provides many kinds of data for researchers in [10], such as the HapMap genotype data and the phased haplotype data. The phased haplotype data describes SNP alleles on a chromosome inherited from one of father and mother, while the genotype data describes SNP alleles on both chromosomes inherited from parents [13]. We give an example (see Fig. 1(a)) to describe the relationship between the haplotype and genotype. Besides, the genotype data has missing values for some loci (SNPs), while the phased haplotype data (also called as the HapMap Phase II haplotypes data) has missing values filled by the well known genotype phasing tool PHASE [14,15]. Therefore, we download the phased haplotype data from the directory of (Index of/downloads/phasing/2006–07 phaseII/phased). The legend data and sample data in the directory are also necessary to describe locus places (feature IDs), locus names (feature names), and sample names (individual IDs). The HapMap data includes four populations: CEU, YRI, JPT and HCB, where CEU represents Utah residents with ancestry from northern and western Europe; YRI represents Yoruba individuals from Ibadan and Nigeria; JPT represents Japanese individuals from Tokyo, and HCB means Han Chinese individuals from Beijing. CEU and YRI each has 90 related samples, i.e., 30 father-mother-offspring trios. After removing the offsprings, 60 unrelated samples are obtained for CEU and also for YRI. For JPT and CHB populations, each of them has 45 unrelated samples. Therefore, we obtain 210 unrelated samples for the experiment. Since the HapMap Project provides 4 separate populations and also 3 populations, we will do the classification on the 3-population and 4-population problems, respectively. The 3-population problem is the same as [7].

Combining all the features together from the 23 chromosomes, i.e., Chromosome 1, 2, ..., 22, X (the phased data of Chromosome Y is not available), we have nearly 4 million SNPs involved in the experiment. For most features (locus), their SNP types (feature values) are expressed as bi-allelic SNPs, i.e., consisting of two single alleles from 4 nucleotides *ATCG*. For example, if one SNP consists of the two allels *A *and *G *(see Fig. 1(b)), all the possible feature values for this locus will be *AA*, *AG *and *GG*, in which *AA *and *GG *are called homozygous, and *AG *is called hyterozygous. Since the phased haplotype data has two rows of haplotypes describing one individual, we transform the haplotype data into the genotype format (see Fig. 1(a)) for computational convenience. When transforming data from the haplotype format into the genotype format, we adopt two kinds of transformations considering different requirements of two algorithms (see Fig. 1(c)). For the modified t-test ranking measure, if simply transforming nominal values to normal numeric values and doing the calculation according to Equation (4), it will be possible to lose the meaning of different SNP types. We propose to use vectors to represent different SNP types and rank them by the modified t-test ranking measure (Equation (5)). For example (see Fig. 1(c)), according to the description of the modified t-test ranking measure, "11" (i.e,, *AA *in Fig. 1(c)) is represented by {0, 0, 1}, "00" (*GG*) is represented by {0, 1, 0}, and "10" (*AG*) is represented by {1, 0, 0}. As to the F-statistics ranking measure, since it involves the calculation of two single alleles for each bi-allelic SNPs, we will use 1 and 0 to represent the two different alleles, respectively. For example, given the same SNP reference type *A/G *as the one in the modified t-test ranking measure, we use 1 to represent *A *and 0 to represent *G*. Then, in each population we can calculate each allele's frequency and variation for each population, as well as those values for all the populations. Each SNP's F-statistics value is calculated from Equation (7). At the same time, we notice some special conditions. For example, if one locus with reference SNP type *A/G *only has the value *AA *for all the individuals, the frequency of the SNP allele *A *will be 100% and the frequency of allele *G *will be zero. Referring to Equation (7), either $\bar{p}$ or $\bar{q}$ in the denominator will be equal to zero. In fact, this feature has no classification capability for any populations. Therefore, we set the *F*_*st *_value of that feature as zero. In summary, the greater the numerator and the smaller the denominator in Equation (7), the greater the value *F*_*st *_and the more important the corresponding feature for classification.

We have 4 simulations to conduct, i.e., 4 different combinations of two rankings (F-statistics and modified t-test) and two classifications (on 3 populations and 4 populations, respectively). From the 210 samples, we randomly choose 40 samples from YRI and CEU, respectively, and 30 samples from JPT and CHB, respectively, as the training set. The 70 samples left are used as the testing set. Each simulation is repeated for 30 times.

We first rank the SNPs of 23 chromosomes, respectively. Then we choose each chromosome's top 100 SNPs, combine the 2300 features together, and rank them again. In this way, features involved in the experiment are greatly reduced and this also will not lead to loss of important information. On the contrary, it will improve the efficiency of the experiment.

In each of 30 simulations, we select top 100 SNPs from the ranking list and form 100 different SNP subsets. The first subset consists of only the first top SNP. The second subset is formed by adding the second top SNP into the previous subset, the third top. Subsequently, we evaluate all subsets through the classifier SVM in terms of the classification accuracy. Due to space limitation, we provide classification results of only 11 feature subsets, i.e., the subsets consisting of 1, 10, 20, 30, 40, 50, 60, 70, 80, 90 and 100 SNPs, respectively. Table 1 is for the F-statistics ranking measure and Table 2 is for the modified t-test ranking measure. Intuitively, the classification on 3 populations produces higher accuracies than on 4 populations, for both ranking measures. This can be interpreted by the fact that the JPT and CHB populations have very similar DNA sequence and it is hence hard to discriminate between these two populations. When comparing classification results by the F-Statistics ranking measure with those by the modified t-test ranking measure on 3 populations, we can see that mean accuracies produced by the latter are higher than those by the former for most SNP subsets. The advantage of the modified t-test measure over the F-statistics measure is more obvious for 4 populations than for 3 populations. In addition, we provide the minimal and maximal accuracies for each of those 11 SNP subsets in the 30 simulations for the two ranking measures in Tables 1 and 2, respectively. According to the results in Tables 1 and 2, we can see that complete classification on 3 populations is possible for the modified t-test ranking measure with appropriate SNP subsets. Whereas, there is always a little error (i.e., 1/70) for the F-statistics measure with those 100 SNP subsets.

**Table 1:** Classification accuracy results obtained by the F-statistics measure for different feature subsets with different numbers of top ranked features (SNPs) in 30 simulations, on 3 and 4 populations, respectively

Feature Numbers	Mean accuracy ± std (minimal/maximal accuracy) (%) for 3 populations	Mean accuracy ± std (minimal/maximal accuracy) (%) for 4 populations
1	69.21 ± 1.60 (64.29/70)	54.98 ± 1.60 (51.43/57.14)
10	72.96 ± 7.82 (64.29/92.86)	56.16 ± 2.36 (45.71/58.57)
20	74.48 ± 7.82 (65.71/95.71)	57.88 ± 4.26 (54.29/74.29)
30	74.92 ± 8.79 (65.71/95.71)	58.47 ± 5.18 (48.57/74.29)
40	77.29 ± 10.55 (65.71/97.14)	59.51 ± 5.08 (54.29/77.14)
50	79.75 ± 11.96 (64.29/98.57)	61.18 ± 7.24 (54.29/82.86)
60	82.46 ± 11.41 (67.14/98.57)	64.09 ± 7.10 (57.14/82.86)
70	84.68 ± 11.15 (67.14/98.57)	64.48 ± 7.73 (57.14/82.86)
80	94.48 ± 7.03 (64.29/98.57)	67.98 ± 8.86 (55.71/84.29)
90	93.74 ± 5.98 (68.57/98.57)	70.84 ± 9.13 (57.14/87.14)
100	93.79 ± 3.44 (80/98.57)	73.99 ± 7.09 (58.57/87.14)

**Table 2:** Classification accuracy results obtained by the modified t-test measure for different feature subsets with differen numbers of top ranked features (SNPs) in 30 simulations, on 3 and 4 populations, respectively

Feature Numbers	Mean accuracy ± std (minimal/maximal accuracy) (%) for 3 populations	Mean accuracy ± std (minimal/maximal accuracy) (%) for 4 populations
1	69.37 ± 1.43 (65.71/71.43)	54.86 ± 1.54 (51.43/57.14)
10	72.97 ± 7.14 (60.00/92.86)	56.29 ± 6.03 (45.71/74.29)
20	75.20 ± 7.82 (65.71/95.71)	58.45 ± 7.15 (48.57/74.29)
30	76.69 ± 9.23 (67.14/95.71)	60.17 ± 9.34 (50.00/81.43)
40	77.03 ± 8.65 (68.57/94.29)	61.60 ± 7.77 (51.43/78.57)
50	79.94 ± 9.36 (55.71/97.14)	65.20 ± 8.21 (54.29/81.43)
60	81.89 ± 11.03 (61.43/100)	69.26 ± 9.21 (51.43/84.29)
70	85.23 ± 10.92 (70.00/100)	70.34 ± 9.38 (52.85/84.29)
80	94.57 ± 9.75 (81.43/100)	73.94 ± 7.73 (58.57/84.29)
90	94.29 ± 3.73 (84.29/98.57)	79.60 ± 4.53 (67.14/87.14)
100	94.57 ± 3.06 (84.29/98.57)	80.46 ± 4.57 (68.57/90.00)

In the following, we find the subset which leads to the maximal classification accuracy from the 100 SNP subsets (see Table 3). We list the maximal classification accuracy in each of the 30 simulations, the number of SNPs that the relevant SNP subset includes, and the mean values (± standard deviations) in the 30 simulations. Although the average number of SNPs that leads to the best classification is similar for both ranking measures (see the 5th column of Table 3), the mean classification accuracies produced by two ranking measures are different (see the 2nd column of Table 3). The modified t-test ranking measure produces 97.09% mean accuracy, which is 1.04% higher than the accuracy produced by the F-statistics measure, i.e., 96.05%, for 3 populations. The mean accuracy produced by the modified t-test on 4 populations, i.e., 83.86%, is much higher than that produced by the F-statistics measure, i.e., 77.34%.

**Table 3:** The maximum classification accuracy in each of 30 simulations together with the mean accuracy (standard deviation), and the relevant feature numbers leading to the maximal accuracy together with the mean number (standard deviation), for 3 populations and 4 populations, respectively

Feature Numbers	Maximum accuracy (%)	Mean accuracy ± std (%)	Relevant feature numbers	Average number of features ± std
f-statistics on 3 populations	94.29 98.57 95.71 97.14 94.29	96.05 ± 1.58 (%)	12 85 15 42 29 86	63.6 ± 25.8
	94.29 95.71 95.71 95.71 97.14		37 56 78 90 71 46	
	97.14 95.71 97.14 95.71 98.57		79 79 49 8 75 74	
	92.86 97.14 97.14 97.14 92.86		74 100 83 50 81	
	97.14 95.71 95.71 95.71 97.14		67 38 82 81 93 84	
	95.71 98.57 95.71 97.14 92.86		53	

f-statistics on 4 populations	78.57 78.57 85.71 70.00 88.57	77.34 ± 6.57 (%)	53 98 82 96 91	85.2 ± 15.1
	78.57 80.00 82.86 70.00 80.00		100 93 81 99 90	
	65.71 78.57 82.86 68.57 81.43		59 99 88 55 73 81	
	80.00 70.00 78.57 81.43 74.29		56 99 100 100 74	
	72.86 84.26 74.29 84.29 88.57		74 98 94 88 72 81	
	68.57 75.71 64.29 75.71 70.00		99 98 90	

Modified t-test on 3 populations	95.71 100.00 95.71 98.57	97.09 ± 1.74 (%)	27 84 19 90 29 95	64.0 ± 26.5
	94.29 98.57 95.71 95.71		64 80 84 80 78 54	
	95.71 98.57 95.71 95.71 98.57		83 92 57 11 80 79	
	100.00 100.00 97.14 98.57		78 95 62 75 94 84	
	95.71 97.14 98.57 98.57 95.71		53 10 32 28 68 55	
	94.29 94.29 97.14 97.14 95.71			
	98.57 97.14 98.57			

Modified t-test on 4 populations	82.86 77.14 87.14 82.86 82.86	83.86 ± 3.16 (%)	31 84 92 94 86	84.1 ± 16.3
	84.29 88.57 80.00 81.43 82.86		100 84 99 83 87	
	82.86 84.29 87.14 84.29 85.71		96 61 83 93 99 95	
	81.43 82.86 84.29 84.29 75.71		80 81 88 99 99 75	
	81.43 84.29 81.43 84.29 90.00		95 82 95 51 83 73	
	84.29 85.71 85.71 85.71 90.00		99 55	

After determining the subset leading to the maximal classification accuracy in each of the 30 simulations, we need further determine what those SNPs are and which chromosomes those SNPs locate on. From the result in Table 3, we know there are on average 64 SNPs obtained for the desirable feature subset. Because of space limitation, we will not list all those SNPs. For example, in Table 4, we list 22 SNPs whose appearance frequencies are greater than 83.33% (i.e., appearing more than 25 times in the 30 simulations), mean ranking values and locations of these SNPs, using the F-statistics ranking measure on 3 populations. Similarly, we present results obtained by the modified t-test ranking measure in Table 5, in which 24 top ranked SNPs whose appearance frequencies are greater than 83.33% in the 30 simulations are presented. For both ranking methods, most of the SNPs come from chromosome 11 (chr11), except rs35397 from the chromosome 5 (chr5), rs2296224 from chromosome 1 (chr1) and rs199138 from chromosome 15 (chr15). Among 22 SNPs in Table 4, rs1604797 and rs7946015 appear 30 times in the 30 simulations. Among 24 SNPs in Table 5, rs1604797, rs7946015 and rs10832001 appear 30 times in the 30 simulations. Interestingly, the mean ranking values of these SNPs with the highest appearance frequencies are not the highest.

**Table 4:** Top ranked features whose appearance frequencies are greater than 83.33% (25/30) in 30 simulations, and their mean ranking values by the F-statistics ranking measure for 3 populations

Ranking No. on Mean Ranking Values	Name of SNPs	Chromosome	Mean ranking values in 30 simulations	Ranking No. on Appearance Frequency
1	rs232045	chr11	0.9573	7
2	rs12786973	chr11	0.9547	6
3	rs7946015	chr11	0.9544	2
4	rs4756778	chr11	0.9524	3
5	rs7931276	chr11	0.9521	9
6	rs4823557	chr11	0.9518	5
7	rs10832001	chr11	0.9506	4
8	rs35397	chr5	0.9491	8
9	rs11604470	chr11	0.9480	12
10	rs10831841	chr11	0.9478	11
11	rs2296224	chr1	0.9456	10
12	rs12286898	chr11	0.9387	13
13	rs1869084	chr11	0.9341	20
14	rs4491181	chr11	0.9307	26
15	rs1604797	chr11	0.9258	1
16	rs7931276	chr11	0.9161	14
17	rs11826168	chr11	0.9103	19
18	rs477036	chr11	0.9072	16
19	rs7940199	chr11	0.9032	22
20	rs4429025	chr11	0.8711	25
21	rs6483747	chr11	0.8435	17
22	rs199138	chr15	0.8417	18

**Table 5:** Top ranked features whose appearance frequencies are greater than 83.33% (25/30) in 30 simulations, and their mean ranking values by the modified t-test ranking measure for 3 populations

Ranking No. on Mean Ranking Values	Name of SNPs	Chromosome	Mean ranking values in 30 simulations	Ranking No. on Appearance Frequency
1	rs232045	chr11	8.0956	7
2	rs1869084	chr11	8.0886	9
3	rs4756778	chr11	8.0079	6
4	rs11218714	chr11	8.0047	11
5	rs10832001	chr11	7.9810	3
6	rs7946015	chr11	7.8988	2
7	rs11826168	chr11	7.8517	4
8	rs704737	chr11	7.7786	18
9	rs1083184	chr11	7.7778	24
10	rs16913196	chr11	7.7774	13
11	rs12786973	chr11	7.7499	5
12	rs12286898	chr11	7.7421	12
13	rs11604470	chr11	7.7401	16
14	rs35397	chr5	7.7257	8
15	rs7931276	chr11	7.7060	14
16	rs477036	chr11	7.6644	17
17	rs6483747	chr11	7.6625	19
18	rs7931276	chr11	7.5996	15
19	rs1604797	chr11	7.3847	1
20	rs10836565	chr11	7.3053	10
21	rs2296224	chr1	7.1358	21
22	rs4275650	chr11	7.0043	23
23	rs7924569	chr11	6.9431	20
24	rs2582905	chr11	6.9264	22

All experiments are executed using Matlab 7.1 on a personal computer with Windows XP operating system and Pentium 4 CPU (3.4 GHZ) and 1 GHZ RAM. We perform statistics about the running time of the two ranking measures together with the training and testing time. The mean time using the F-statistics to rank all SNPs of 3 populations is 5342.9 seconds, while on average 5728.7 seconds for the modified t-test ranking measures. It may be because that calculating the median value *S*_0 _makes the modified t-test ranking measure take more time than the F-statistics measure. Both algorithms cost more time on 4 populations compared to 3 populations. The total training and testing time is 6915.1 seconds. In terms of classification accuracy, the modified t-test ranking measure is superior over the F-statistics measure. Besides, the modified t-test ranking measure is proposed to deal with vector features and provides a way for ranking nominal features.

Since features' ranking only indicates the relevance of each feature, those features with the same or close ranking values may have high correlation between each other, i.e., redundancy. Therefore, it is possible for us to further reduce the number of SNPs in our future work.

## Conclusion

In this paper, we propose to use two feature importance ranking measures, i.e., the modified t-test and F-statistics, to rank large amount of SNPs, and then use the greedy manner together with a classifier to determine a desirable feature subset, which has the minimum size but leads to the highest classification accuracy. The final results show that both ranking methods are efficient on determining the importance of the SNPs. Although the two ranking measures find nearly the same amount of SNPs, the modified t-test ranking measure tends to be better than the F-statistics measure in terms of the classification accuracy. Compared to the classification method of Park *et al.*[7], we obtain a better result, i.e., good classification of the 3 populations using fewer, i.e., on average 64, SNPs.

## Methods

In classification on large data sets, feature selection is necessary and shows many advantages such as saving computational time, reducing computational burden and improving efficiency. Feature ranking, as an usual step in many feature selection methods [16,17], is adopted in our experiment to determine the features' classification power. In this paper, we will present two feature importance ranking measures: a modified t-test from [9,18,19] and F-statistics [20], and make an comparison about their ranking abilities so as to evaluate the modified t-test ranking measure.

### Modified T-test

The original t-test, i.e., the student t-test [18], can be used to evaluate whether the means of two classes are statistically different from each other by calculating a ratio between the difference of two class means and variability of the two classes. It has been adopted by [21,22] to rank features (genes) for microarray data and for mass spectrometry data [23,24]. We notice that the original t-test is only applied on 2-class problems. In the following multi-class problems, Tibshirani *et al.*[9] developed the nearest shrunken centroid method, i.e., calculating a t-statistic value (Equations (1)) for each gene of each class. This t-statistic value measured the difference between the mean of one class and the mean of all the classes, and the difference is standardized by the within-class standard deviation.

$$t_{ic}=\frac{{\bar{x}}_{ic}-{\bar{x}}_{i}}{M_{c}\cdot (S_{i}+S_{0})}$$

$$S_{i}^{2}=\frac{1}{N-C}\sum_{c=1}^{C}\sum_{j\in c}{\left(x_{ij}-{\bar{x}}_{ic}\right)}^{2}$$

$$M_{c}=\sqrt{1/n_{c}+1/N}$$

Here *t*_*ic *_indicates the t-statistics value for the *i*-th feature of the *c*-th class. ${\bar{x}}_{ic}$ indicates the *i*-th feature's mean value in the *c*-th class and ${\bar{x}}_{i}$ indicates the *i*-th feature's mean value for all classes. *x*_*ij *_represents the *i*-th feature of the *j*-th sample. *N *is the total number of all the samples for all the *C *classes and *n*_*c *_is the number of samples for the *c*-th class. *S*_*i *_is the within-class standard deviation and *S*_0 _is set to be the median value of *S*_*i *_for all the features. This t-statistic value of Tibshirani et al. [9] measured the deviation between each class and the mean of all classes and was used to constitute a classifier. The authors did not refer to using the t-statistic of each class to rank features for all the classes. In [19], Wang et al. extended the t-statistic algorithm to rank features for all the classes. That is, the t-score (t-statistic value) of feature *i *is calculated as the greatest t-score for all classes:

$$t_{i}=max\left\{\frac{\left|{\bar{x}}_{ic}-{\bar{x}}_{i}\right|}{M_{c}S_{i}},c=1,2,...C\right\}$$

Due to the characteristic of the SNP data [10], i.e., with nominal values for each feature (e.g., *AA*, *AT *and *TT*), Equation (4) can not be used to deal with our problem. We proposed a modified t-test ranking method, in which different nominal values are represented by different vectors to realize the calculation. In the following, we generalized the t-score of each feature in 3 steps:

1. Suppose the feature set is *F *= (*f*_1_,...,*f*_*i*_, ..., *f*_*g*_), and feature *i *has *m*_*i *_different nominal values represented as $f_{i}=(x_{i}^{\left(1\right)},x_{i}^{\left(2\right)},...,x_{i}^{\left(m_{i}\right)})$

2. Transform each nominal feature value into a vector with the dimension *m*_*i*_, i.e., $x_{i}^{\left(1\right)}\Rightarrow X_{i}^{\left(1\right)}=\{0,...,0,1\},x_{i}^{\left(2\right)}\Rightarrow X_{i}^{\left(2\right)}=\{0,...,1,0\},...,x_{i}^{\left(m_{i}\right)}\Rightarrow X_{i}^{\left(m_{i}\right)}=\{1,...,0,0\}$.

3. Replace all the numerical features in Equations (1) and (2) with those vectors (see Equations (5) and (6)).

$$t_{i}=max\left\{\frac{\left|{\bar{X}}_{ic}-{\bar{X}}_{i}\right|}{M_{c}S_{i}},c=1,2,...C\right\}$$

$$S_{i}^{2}=\frac{1}{N-C}\sum_{c=1}^{C}\sum_{j\in c}(X_{ij}-{\bar{X}}_{ic}){\left(X_{ij}-{\bar{X}}_{ic}\right)}^{T}$$

The ranking rule is: the greater the t-scores, the more relevant the features.

### F-statistics

In our experiment, we will use another ranking measure, i.e., F-statistics, to make a comparison with the modified t-test. The version of F-statistics used in our experiment is based on the definition of [25], which was originally developed by [20] and used in population genetics to describe the level of heterozygosity in a population.

Given a SNP genotype data with *C *sub-populations and each feature expressed as bi-allelic SNPs (i.e., consisting of any two different nucleotides from the four nucleotides *ATCG*), the F-statistics (*F*_*st*_) is calculated as:

$$F_{st}=Var(p)/(\bar{p}*\bar{q})$$

where *p *and *q *are corresponding to the two alleles' frequencies, respectively, in one population. $\bar{p}$ and $\bar{q}$ refer to the two alleles' mean frequencies for all the population classes. *Var*_*p *_represents the variance (See Equation 8) of one allele.

$$Var(p)=\sum_{c=1}^{C}{\left(p_{c}-\bar{p}\right)}^{2}/C$$

Here, *p*_*c *_designates the frequency of one allele for the *c*-th population. And the mean frequency is easy to obtain from:

$$\bar{p}=\sum_{c=1}^{C}p_{c}$$

The ranking rule is same as the modified t-test, i.e., the larger the *F*_*st *_value, the more significant the SNP for population classification.

### The Classifier

Although many classifiers, such as classical neural network, naive Bayes classifier and so on, can be applied in our classification, here we would like to choose the support vector machine (SVM) [11,12] in our experiment because of its some attractive features, such as effectively avoiding overfitting and accomodating large feature spaces, fast speed and so on. It will be used not only in the final classification, but also in the feature selection to test different feature subsets and determine the one with the highest classification accuracy. During the classification process, we determine the kernel parameter *γ *and the penal parameter *ν *through the double cross-validation method.

## Authors' contributions

LW proposed to use statistic ranking methods to select informative SNPs on the HapMap genotype data for population classification. NZ conducted the algorithm implementations and drafted an early version of the manuscript. LW revised the draft.
